# Psilocybin Maintains Better Brain Function in an Alzheimer’s Disease Model with Reduced Neuroinflammation and Improved Hippocampal Neurogenesis

**DOI:** 10.1002/alz70859_100589

**Published:** 2025-12-25

**Authors:** Leelavathi N Madhu, Yogish Somayaji, Sanya Kotian, Sahithi Attaluri, Jay Patel, Prashanta K Panda, Vidya Rao, Maheedhar Kodali, Goutham Shankar, Shama Rao, Susan Weintraub, Bing Shuai, Xiaolan Rao, Ashok K Shetty

**Affiliations:** ^1^ Institute for Regenerative Medicine, Department of Cell Biology and Genetics, Texas A&M University College of Medicine, Bryan/College Station, Texas, USA., College Station, TX USA; ^2^ Institute for Regenerative Medicine, Department of Cell Biology and Genetics, School of Medicine, Texas A&M University Health Science Center, College Station, Texas, USA., College Station, TX USA; ^3^ Institute for Regenerative Medicine, Dept of Cell Biology and Genetics, Texas A&M Univ School of Medicine, College Station, TX USA; ^4^ Department of Biochemistry and Structural Biology, UT Health San Antonio, San Antonio, Texas, USA, San Antonio, TX USA

## Abstract

**Background:**

Chronic neuroinflammation plays a significant role in Alzheimer’s disease (AD) pathogenesis associated with a decline in cognitive and mood function. Currently, there are no effective therapies to alleviate the progression of brain dysfunction in AD. Psilocybin, an FDA‐approved drug for treating major depressive disorder, can restrain neuroinflammation and improve hippocampal neurogenesis. Therefore, the current study investigated the efficacy of psilocybin treatment in slowing down cognitive decline in 5x familial AD (5xFAD) mice.

**Methods:**

Three‐month‐old male 5xFAD mice received monthly psilocybin (0.5mg/Kg) or vehicle treatment for 4 months (AD‐Psilocybin and AD‐Veh groups). A month after the last dose of psilocybin, the animals were interrogated with neurobehavioral tests to ascertain the extent of cognitive and mood function decline compared to age‐matched naïve control mice. The animals were euthanized when they were 8 months old, and brain tissues were analyzed for the extent of neuroinflammation, hippocampal neurogenesis, synapse loss, and amyloid‐beta plaques. The hippocampi from AD‐Psilocybin and AD‐Veh groups were also analyzed using proteomics.

**Results:**

Mice in the AD‐Psilocybin group displayed improved abilities to discern minor changes in the immediate environment, pattern separation, associative recognition memory, and no anhedonia compared to mice in the AD‐Veh group. Analyses of brain tissues revealed a significant reduction of chronic neuroinflammatory markers in the AD‐Psilocybin group vis‐à‐vis the AD‐Veh group. These were apparent from reductions in astrocytic hypertrophy, microglial inflammasome complexes, concentrations of mediators and end products of NLRP3 inflammasome activation, proteins involved in p38/mitogen‐activated protein kinase hyperactivation, and proteins linked to activation of cGAS‐STING signaling. Additionally, AD‐Psilocybin mice exhibited increased production of new neurons associated with improved maintenance of BDNF‐ERK‐CREB signaling and synaptic proteins. Proteomic analysis of the hippocampus revealed the upregulation of 16 proteins involved in regulating neuroinflammation, mTOR signaling, synaptic function, and axon extension in the AD‐Psilocybin group. Notably, mice in the AD‐Psilocybin group did not exhibit reduced amyloid‐beta plaques or the formation of microglial clusters around plaques.

**Conclusions:**

Psilocybin treatment can maintain better brain function in an AD model without affecting amyloid‐beta plaques. Improved brain function is likely due to psilocybin‐induced reductions in neuroinflammatory signaling, enhanced hippocampal neurogenesis, and preservation of synapses.

